# Cruciferous vegetable intake is inversely associated with lung cancer risk among smokers: a case-control study

**DOI:** 10.1186/1471-2407-10-162

**Published:** 2010-04-27

**Authors:** Li Tang, Gary R Zirpoli, Vijayvel Jayaprakash, Mary E Reid, Susan E McCann, Chukwumere E Nwogu, Yuesheng Zhang, Christine B Ambrosone, Kirsten B Moysich

**Affiliations:** 1 Department of Cancer Prevention and Control, Roswell Park Cancer Institute, Elm and Carlton Streets, Buffalo, NY 14263, USA; 2 Department of Medicine, Roswell Park Cancer Institute, Elm and Carlton Streets, Buffalo, NY 14263, USA; 3 Department of Thoracic Surgery, Roswell Park Cancer Institute, Elm and Carlton Streets, Buffalo, NY 14263, USA

## Abstract

**Background:**

Inverse associations between cruciferous vegetable intake and lung cancer risk have been consistently reported. However, associations within smoking status subgroups have not been consistently addressed.

**Methods:**

We conducted a hospital-based case-control study with lung cancer cases and controls matched on smoking status, and further adjusted for smoking status, duration, and intensity in the multivariate models. A total of 948 cases and 1743 controls were included in the analysis.

**Results:**

Inverse linear trends were observed between intake of fruits, total vegetables, and cruciferous vegetables and risk of lung cancer (ORs ranged from 0.53-0.70, with P for trend < 0.05). Interestingly, significant associations were observed for intake of fruits and total vegetables with lung cancer among never smokers. Conversely, significant inverse associations with cruciferous vegetable intake were observed primarily among smokers, in particular former smokers, although significant interactions were not detected between smoking and intake of any food group. Of four lung cancer histological subtypes, significant inverse associations were observed primarily among patients with squamous or small cell carcinoma - the two subtypes more strongly associated with heavy smoking.

**Conclusions:**

Our findings are consistent with the smoking-related carcinogen-modulating effect of isothiocyanates, a group of phytochemicals uniquely present in cruciferous vegetables. Our data support consumption of a diet rich in cruciferous vegetables may reduce the risk of lung cancer among smokers.

## Background

Lung cancer is the second most common cancer, and represents the leading cause of cancer death in both men and women. An estimated 29% of all cancer deaths in 2008 in the United States was expected from lung cancer [1]. Due to the poor survival rate, prevention has been the primary focus in fighting lung cancer. Cigarette smoking is the leading risk factor for lung cancer, contributing to 80-90% of lung cancer cases [2]. The primary public strategy to prevent lung cancer is to prevent initiation and promote cessation of smoking. In addition, certain dietary components may also be able to contribute to the prevention or delay of lung cancer among smokers by modifying the carcinogenic effects of cigarette smoking. Of particular interest are cruciferous vegetables which are rich sources of glucosinolates, the precursors of isothiocyanates as well as indole-3-carbinol. Isothiocyanates modulate carcinogen metabolism and facilitate carcinogen detoxification via altering phase 1 and phase 2 enzyme systems, thus inhibiting carcinogenesis [3-5]. Inhibition of tobacco-related carcinogenesis is the most thoroughly studied example of this effect by isothiocyanates [6,7]. Using NNK, a tobacco-specific nitrosamine carcinogen, as an example, it has been shown that isothiocyanates potently inhibit NNK-induced lung carcinogenesis in animal models, which is closely correlated with their ability to inhibit NNK-activating phase 1 enzymes [8,9]. In a group of smoking volunteers, Hecht et al. showed that consumption of watercress, a cruciferous vegetable and a rich source of phenethyl isothiocyanate, leads to inhibition of oxidative metabolism of NNK, and increase of urinary excretion of NNK metabolites [10]. Further, London et al. reported that individuals with detectable urinary isothiocyanate levels were at a lower risk of lung cancer in a cohort study in China (RR = 0.65, 95% CI = 0.43-0.97) [11].

Cohort and case-control studies have demonstrated negative associations between cruciferous vegetable consumption and lung cancer [12,13]. However, associations within smoking status subgroups have not been consistently addressed. Gao et al. reported that cabbage consumption in Japan was inversely associated with lung cancer risk among current smokers (OR = 0.3) [14]; however, this inverse association was found only among former smokers (OR = 0.4) in the Iowa Women's Health Study [15]. Koo et al. reported no association between brassica consumption and lung cancer risk among female nonsmokers of Hong Kong [16]. Smoking is strongly associated with lung cancer risk and smokers tend to eat fewer vegetables and fruits than nonsmokers do [17,18]. Importantly, some of these studies adjusted only for either smoking status or pack-years of smoking, which may not adequately control the confounding effect of smoking. These limitations might contribute at least in part to the observed inconsistencies.

In the current study, cases and controls were matched on smoking status, and then smoking status as well as smoking duration and intensity were further adjusted in the multivariate models, in an attempt to decrease the degree of residual confounding by cigarette smoking and help to delineate the effect of cruciferous vegetables on lung cancer risk in relation to smoking. Moreover, unlike previous studies in which only one or several kinds of cruciferous vegetables were examined to represent consumption of cruciferous vegetables, the current study included almost all the cruciferous vegetables commonly consumed in the source population (a total of eight types) from which the study sample was derived. More importantly, this is the first study to consider raw versus cooked cruciferous vegetable intake in relation to lung cancer risk. It has been known that isothiocyanate yield can be greatly reduced by cooking procedures [19-22]. In this hospital-based case-control study, we examined the association between raw and total cruciferous vegetable intake and lung cancer risk by smoking status as well as among histological subtypes.

## Methods

### Study population and case-control selection

Data used in this study were collected from individuals receiving medical services at Roswell Park Cancer Institute (RPCI) between 1982 and 1998 who participated in the Patient Epidemiology Data System (PEDS) study. Participants were predominantly Caucasian (94%) and ranged from 26 to 90 years of age. The cases included 948 individuals diagnosed with primary, incident lung cancer, who were identified from the RPCI tumor registry and Diagnostic Index with the following histologies: squamous cell carcinomas (37%), small cell carcinomas (22%), large cell carcinomas (17%), adenocarcinomas (13%), and others (11%). Controls were frequency matched to cases on 5-y age strata, gender, smoking status, and decade in which they completed the questionnaire with an attempted control-to-case ratio of 2:1. However, because of the relatively stringent matching criteria, fewer than 2 controls per case were available in certain strata. A total of 1743 controls were included in the analysis, selected from a pool of 7845 potentially eligible controls. These participants came to RPCI with a suspicion of neoplastic disease, but were diagnosed with conditions other than cancer, including diseases of the gastrointestinal system (24%), the genitourinary system (20%), and the circulatory system (12%), infectious and parasitic diseases (15%), and other conditions (29%) during the same time period as were cases. Cruciferous vegetable intake did not significantly differ by disease diagnosis among the controls, suggesting that disease status among controls was not associated with dietary pattern. The RPCI Institutional Review Board approved the conduct of the study and all participants provided informed consent.

### Questionnaire

The PEDS questionnaire was used to collect data, and was offered to all new patients receiving medical service at RPCI between 1982 and 1998 as part of the admission process. The overall response rate for both controls and cases was ~50%. The questionnaire requested detailed information on demographic background, occupational and environmental exposures, tobacco and alcohol consumption, reproductive experiences, medical history, and family history of cancer, and as well as a 44-item food frequency questionnaire (FFQ) assessing usual diet in the few years before diagnosis. Although brief, the 44-item FFQ was designed to provide an assessment of intakes of fruits and vegetables, cruciferous vegetables, and foods providing good sources of vitamins A, C, and E, fat, and fiber [23].

The cruciferous vegetables queried included broccoli (raw and cooked), cabbage (raw and cooked), cauliflower (raw and cooked), Brussels sprouts, and greens including kale, turnip, collard, and mustard greens. Raw cruciferous vegetables as a group were defined as raw broccoli, cabbage, and cauliflower to better represent dietary exposure to isothiocyanates. Questions about cigarette smoking included smoking status (never, former, current), year and age at onset and at quitting, years of smoking, and number of cigarettes smoked per day. Smokers who quit smoking for at least six months were considered former smokers.

### Statistical analysis

Monthly frequency of use for each food category (fruits, vegetables, cruciferous vegetables, raw cruciferous vegetables), was calculated as the sum of the monthly frequency of use of the member food items on the PEDS questionnaire. Intake for each category was divided into quintiles/tertiles based on the distribution of intake in the controls. For individual cruciferous vegetables, intake was divided into two categories (<1, or = 1 servings per month, 1 serving = 1/2 cup) for the purpose of comparison among vegetables. For stratified analyses, smoking duration and intensity were dichotomized at 30 years of smoking and 20 cigarettes per day, respectively, on the basis of distribution in ever-smoking controls (including both former and current smokers).

Two-tailed *t*-test and Pearson's χ^2 ^were conducted to evaluate differences between case and control groups for continuous and categorical variables, respectively. As cases and controls were frequency-matched instead of exact-matched, odds ratios (OR) and 95% confidence intervals (CI) for the association between food group and lung cancer risk were computed using unconditional logistic regression in separate models with the lowest category as the referent [24]. Adjusted logistic models included age (continuous), education level (<high school or >high school), gender (male or female), total meat intake (continuous), smoking status (never, former, or current), number of cigarettes per day (continuous), years of smoking (continuous), and year of admission (continuous). Total meat intake was included in the model to partially adjust for differences in diet quality associated with vegetable intake. In stratified analysis, smoking status, number of cigarettes per day, years of smoking were mutually adjusted. Multinomial logistic regression was used to compare each histological subtype with the controls for each food group. We examined five discrete, histological subtypes: 1) squamous cell carcinoma, 2) small cell carcinoma, 3) large cell carcinoma, 4) adenocarcinoma, 5) others including adenosquamous cell carcinoma, sarcoma and other. The overall ORs and 95% CIs compared all cases combined with controls. Tests for trend were conducted by using the median values for each quintile as continuous variables in the logistic regression models. Multiplicative interactions were tested through inclusion of the cross-product terms in the logistic regression models. Statistical analyses were performed by using SAS for Windows, version 9.1. All tests were two-sided and considered statistically significant at p < 0.05.

## Results

In Table 1 are the descriptive characteristics of cases and controls. No significant differences were observed between cases and controls for age (p = 0.43), indicating the successful matching on age. However, despite the matching on smoking status, mean smoking intensity and duration were significantly different between cases and controls. Among both former and current smokers, compared to controls, cases smoked more cigarettes per day (29.3 versus 22.6, p < 0.0001) and accumulated more years of smoking (37.0 versus 27.1, p < 0.0001). On the other hand, compared to cases, controls consumed more fruits, vegetables, and both raw and total cruciferous vegetables. On average, lung cancer cases also consumed more meats and had lower levels of education. A significant difference in the distribution of year of admission was observed between cases and controls, with more controls than cases enrolled before 1990 (59.1% versus 54.1%, p = 0.017). However, stratification by year of admission in the analyses did not affect our results (results not shown).

**Table 1:** Descriptive Characteristics of Lung Cancer Cases and Hospital Controls.

	Lung cancer cases (N = 948)		Controls (N = 1743)		P
		
	Means ± SD		Means ± SD		(t-test)
	
Age, yr	62.5 ± 10.1		62.2 ± 10.3		0.4322
Vegetables, servings/mo	71.5 ± 41.0		79.6 ± 43.9		<0.0001
Fruits, servings/mo	41.7 ± 32.7		47.8 ± 32.3		<0.0001
Meats, servings/mo	34.8 ± 24.4		30.3 ± 22.1		<0.0001
Cruciferous^1^, servings/mo	12.9 ± 13.7		15.7 ± 16.4		<0.0001
Raw cruciferous^2^, servings/mo	4.2 ± 5.9		5.7 ± 7.9		<0.0001
Cigarettes per day	29.3 ± 15.9		22.6 ± 14.8		<0.0001
Years of smoking	37.0 ± 14.4		27.1 ± 15.6		<0.0001
	**Lung cancer cases**		**Controls**		**P**
		
	**Number**	**%**	**Number**	**%**	**(χ^2^-test)**
	
Education					
< High school	361	38.1	427	24.5	
> High school	587	61.9	1316	75.5	<0.0001
Year of Admission					
1981-1989	514	54.2	1028	59.0	
1990-1999	434	45.8	715	41.0	0.0171
Gender					
Male	583	61.5	1036	59.4	
Female	365	38.5	707	40.6	0.2971
Smoking Status					
Never Smokers	51	5.4	100	5.7	
Former Smokers	684	72.1	1269	72.8	
Current Smokers	213	22.5	374	21.5	0.7930

^1 ^Cruciferous vegetables = broccoli (raw and cooked), cabbage (raw and cooked), cauliflower (raw and cooked), Brussels sprouts, kale, turnip, collard, mustard greens.

^2 ^Raw cruciferous vegetables = raw broccoli, raw cabbage, raw cauliflower.

ORs and 95% CIs for the associations between lung cancer and monthly intakes of fruits, total vegetables, cruciferous vegetables, and raw cruciferous vegetables are presented in Table 2. In unadjusted models, lung cancer risk tended to decrease with increasing consumption of each of these food groups. After adjustment for age, gender, education level, total meat intake, smoking status, number of cigarettes per day, years of smoking, and year of admission, these differences were slightly attenuated but remained statistically significant, with p for trend less than 0.05. Compared to the lowest quintile, the highest quintile of vegetable intake was associated with a 47% reduction in lung cancer risk (OR = 0.53, 95% CI 0.40-0.72). Similarly, a 30% risk reduction was observed with the highest versus lowest quintile of fruit consumption (OR = 0.70, 95% CI 0.52-0.94). Consumptions of both raw and total cruciferous vegetables were also associated inversely with lung cancer risk: those in the highest quintile of consumption had a greater than 40% lower risk compared to those in the lowest quintile (OR = 0.58, 95% CI 0.44-0.79 and OR = 0.59, 95% CI 0.44-0.78, respectively).

**Table 2:** Odds Ratios (OR) and 95% Confidence Intervals (CI) for the Association of Lung Cancer Risk with Fruit, Vegetable, and Cruciferous Vegetable Intake

	Number		Adjusted OR* (95% CI)
		
	Cases	Controls	
Vegetables, servings/mo			
<46	270	351	1.00
46-63	194	349	0.78 (0.60-1.01)
63.5-82	169	348	0.66 (0.50-0.87)
82.5-109.5	182	355	0.76 (0.58-1.00)
>109.5	133	340	0.53 (0.40-0.72)
			*P = 0.0001*
Fruits, servings/mo			
<19.5	279	362	1.00
19.5-35	189	341	0.70 (0.54-0.91)
35.5-52.5	186	357	0.81 (0.62-1.06)
53-71.5	144	336	0.65 (0.49-0.87)
>71.5	150	347	0.70 (0.52-0.94)
			*P = 0.0172*
Cruciferous, servings/mo			
<5	280	383	1.00
5-7.5	181	324	0.85 (0.65-1.11)
8-13.5	198	339	0.87 (0.67-1.13)
14-25	159	359	0.70 (0.53-0.92)
>25	130	338	0.59 (0.44-0.79)
			*P = 0.0002*
Raw cruciferous, servings/mo			
<1	297	405	1.00
1-2	203	303	0.95 (0.73-1.23)
2.5-4	180	362	0.73 (0.56-0.94)
4.5-10	141	325	0.72 (0.55-0.96)
>10	127	348	0.58 (0.44-0.78)
			*P = 0.0002*

* Odds ratios and 95% confidence intervals were calculated with unconditional logistic regression adjusted for age (continuous), education level (<high school or >high school), gender (male or female), total meat intake (continuous), smoking status (never, quit, or current), number of cigarettes per day (continuous), years of smoking (continuous), and year of admission (continuous).

To examine lung cancer risk with cruciferous vegetable intake in relation to smoking, we stratified our analyses by smoking status, smoking intensity (number of cigarettes per day), and smoking duration (years of smoking), mutually adjusted in the logistic regression models. As shown in Additional File 1, both vegetable and fruit intake were significantly inversely associated with lung cancer risk in never smokers; however, the significant inverse associations for both raw and total cruciferous vegetable were observed among smokers only. Among former smokers, an inverse trend was observed with intake of both raw and total cruciferous vegetables (p for trend = 0.01). However, among current smokers, only raw cruciferous vegetable intake was significantly associated with lung cancer risk for the lowest tertile compared to the middle tertile (OR = 0.50, 95% CI 0.31-0.79) but not with the highest tertile (OR = 0.73, 95% CI 0.45-1.18). Lack of statistical power due to fewer patients in the highest tertile may have partly contributed to this phenomenon. Among never smokers, no associations were observed with intake of either raw or total cruciferous vegetables. However, the multiplicative interactions between cruciferous vegetable intake and lung cancer risk were not statistically significant (results not shown).

We combined former and current smokers and further stratified by number of cigarettes per day and years of smoking (Additional File 2). In agreement with the above findings, there was no statistically significant association between lung cancer risk and fruit intake. However, for total vegetables, cruciferous vegetables, and raw cruciferous vegetables, significant inverse associations were observed among heavy smokers (greater than 20 cigarettes per day). Those heavy smokers in the highest tertile versus lowest tertile of intake had a 32% to 48% reduction in lung cancer risk. When stratified with years of smoking, stronger associations for each category were observed in those with 30 years or less of smoking. The strongest association was observed with intake of raw cruciferous vegetables. Compared to those who consumed less than 2.5 servings per month, consumers of greater than 4.5 servings of raw cruciferous vegetables per month had a 55% reduction in lung cancer risk (OR = 0.45, 95% CI 0.30-0.68). We also explored interactions between intake of each food category and smoking behavior in relation to lung cancer risk. No significant interactions were observed, except for total vegetable intake and years of smoking (p = 0.0221). As shown in Additional file 2, the inverse association between vegetable intake and lung cancer risk was statistically significant in those with 30 years or less of smoking, but not among the long-term smokers (> 30 years of smoking).

Associations for all lung cancer combined and by histological subtype with monthly intake of vegetables and fruits are shown in Additional File 3. Among squamous and small cell carcinoma only and consistently across total vegetables, cruciferous vegetables, and raw cruciferous vegetables, higher consumption was associated with lower risk with significant p for trend. No association was found with other subtypes of lung cancer. Compared to 2.5 servings per month of raw cruciferous vegetables, consumption of 4.5 servings per month was significantly associated with development of squamous cell carcinoma with a 42% reduction in the risk (95% CI 0.42-0.80), and a 51% reduction in the risk was observed for small cell carcinomas (95% CI 0.32-0.74). No associations were observed for fruit consumption with any subtype of lung cancer.

## Discussion

Consistent with some previous studies, consumption of fruits, vegetables, and cruciferous vegetables, showed statistically significant inverse associations with lung cancer risk [12,13,25-28]. In our study, intake of total vegetables was inversely associated with lung cancer risk in both never smokers and former smokers. However, when cruciferous vegetables were excluded from total vegetables, the associations were markedly attenuated and no longer statistically significant among former smokers, but the significant associations remained among never smokers (results not shown). Similarly, intake of fruits showed a marginally negative association with lung cancer risk only among never smokers, but no association was observed for ever-smokers, including both former and current smokers. In contrast to total vegetables and fruits, a strong inverse association between cruciferous vegetable intake and lung cancer risk was observed among smokers, in particular former smokers, although we did not detect significant interactions between cruciferous vegetable intake and smoking status. There was no statistically significant association between cruciferous vegetable intake and lung cancer risk among never smokers. Limited power due to relatively small number of never smokers in the current study could contribute to this observation, although this result is consistent with the findings from studies conducted in never smokers [16,29-31].

Our findings indicate that cruciferous vegetables may play a preventive role in lung cancers that are smoking-related, rather than the more general effect of other vegetables and fruits that may be overshadowed by the strong effect of smoking. This hypothesis is supported by the carcinogen-modulating activity of isothiocyanates, a group of natural phytochemicals uniquely present in cruciferous vegetables. Modulation of metabolism of smoking-related carcinogens by isothiocyanates has been documented in both *in vivo *and *in vitro *studies [6,7], as well as in humans [10]. Isothiocyanates have also been shown to inhibit lung tumorigenesis induced by tobacco-specific carcinogens in animal models [9,32,33]. Interestingly, we observed a stronger inverse association between cruciferous vegetable intake and lung cancer risk in the short-term smokers (≤ 30 years of smoking) than the long-term smokers (> 30 years of smoking). Carcinogenesis is an accumulation of carcinogen-induced DNA damage over a long period of time. Although intake of raw cruciferous vegetables/isothiocyanates is able to considerably attenuate smoking-related carcinogen exposure and delay this process significantly, the capacity is not unlimited. Even in the presence of high cruciferous vegetable intake, smokers may still develop lung cancer, especially if they continuously smoke for a long enough time. This is also in line with the finding that the strongest association with intake of cruciferous vegetables was observed among former smokers, instead of current smokers. Comparison of years of smoking between them reveals a longer history of smoking in current smokers (40.3 years) than in former smokers (30.0 years). Furthermore, smoking cessation may represent a healthy life style change possibly accompanied by an increase in vegetable intake. Therefore, smoking cessation should still be emphasized for lung cancer prevention.

Not surprisingly, compared to total cruciferous vegetables, intake of raw cruciferous vegetables, in general, was more strongly inversely associated with lung cancer risk. Isothiocyanates occur naturally in cruciferous vegetables as the precursor glucosinolates (β-thioglucoside N-hydroxysulfates), and their release requires the enzyme myrosinase (thioglucoside glucohydrolase). In the plant, this enzyme is stored physically separated from glucosinolates, and released to hydrolyze glucosinolates when plant cells are damaged. Isothiocyante yield is two to nine times higher from consumption of raw cruciferous vegetables compared with consumption of their cooked counterparts, due to heat-inactivation of myrosinase, destruction of heat-labile isothiocyanates, and loss of glucosinolates during cooking procedures [19-21]. We have previously reported inverse associations between consumption of raw cruciferous vegetables and bladder cancer, another cigarette smoking-related cancer [22]. It is noteworthy that, in addition to isothiocyanate-precursor glucosinolates, cruciferous vegetables also contain other glucosinolates such as indole-precursors. The type and total content of glucosinolates differ substantially among different cruciferous vegetables as well as within the same vegetable under different culture conditions [34]. It is likely that indoles and other phytochemicals and nutrients in cruciferous vegetables such as carotenoids, vitamin C, folic acid, selenium, may also play a role in the chemoprevention of lung cancer, whether or not in combination with isothiocyanates.

Lung cancers were classified into five histological subtypes: small cell carcinoma, squamous cell carcinoma, adenocarcinoma, large cell carcinoma, and the others. When we examined the risk associations among these histological subtypes, significant inverse associations were apparent only among patients with squamous or small cell carcinoma. Interestingly, both squamous and small cell carcinomas tend to be more strongly associated with heavy smoking than other subtypes [35]. Indeed, we found that patients with squamous and small cell carcinoma smoked more (30 cigarettes per day) and longer (39 years of smoking) than did patients with adenocarcinoma (23 cigarettes per day and 29 years of smoking). Additionally, in terms of smoking behavior, patients with adenocarcinoma were more similar to the controls (23.3 versus 22.6 cigarettes per day and 29.4 versus 27.1 years of smoking) (results not shown). Finally, adenocarcinoma occurs in a great proportion of lung cancers in nonsmokers, suggesting factors other than smoking may also play an important role in the etiology of this subtype [36]. Since the potential beneficial role of cruciferous vegetable intake may at least partially rely on modulation of carcinogens in cigarette-smoking by isothiocyanates, it is not surprising to detect differential effects among subtypes of lung cancer.

Several methodological issues should be considered in the interpretation of the results from a hospital-based case-control study. Recall bias is always a concern in case-control studies, as controls may be more motivated than cases to recall cruciferous vegetable intake. However, this may be less of an issue in the current study due to the use of hospital controls who also came to hospital with a suspicion of cancer, but were diagnosed with other diseases. Selection bias may also occur. Both cases and controls were limited to individuals who came to RPCI, a large regional comprehensive cancer center, and may not fully represent the general population or the majority of lung cancer patients. Furthermore, only about 50% of the eligible cases and controls agreed to complete the questionnaire. We were unable to assess whether or not participants and non-participants differed with respect to cruciferous vegetable intake. However, daily intake of cruciferous vegetables among controls in our study (median intake 26.7 g/day, accounting for 14% of all vegetable intake) is comparable to other studies conducted in North America area (daily intake ranges from 16 to 40 g with 5.6 to 15.4% of total vegetable intake) [37], suggesting the detected associations might apply in general population. Misclassification of smoking status might affect our study, as our analysis was based on self-reported data. However, several related questions were employed in the questionnaire to address the same issue, which were highly correlated and would minimize the potential for this source of bias.

The strength of the current study is the use of two approaches to control the confounding effects of smoking: cases and controls were first matched on smoking status; and then smoking status, smoking intensity (indicated by number of cigarettes per day), and smoking duration (indicated by years of smoking) were further adjusted in the data analysis. The need of these approaches is supported by the results shown in Table 1. Even after matching on smoking status, cases still had significantly higher number of cigarettes per day and more years of smoking than controls. These results also indicate that the confounding effect of cigarette smoking may not be adequately controlled by adjusting only for either smoking status or pack-years of smoking, a strategy used in many previous studies. On the other hand, because we were unable to control for the type of cigarettes smoked, pipe and cigar smoking, environmental smoking exposure, residual confounding by smoking may still remain in the current study, albeit to a lesser extent.

## Conclusions

In conclusion, we observed significant linear inverse associations between intake of fruits, total vegetables, and cruciferous vegetables and risk of lung cancer. Contrary to that observed for fruit and vegetable intake which was either unassociated or weakly associated with lung cancer risk among smokers, increasing cruciferous vegetable intake was significantly associated with reduction of lung cancer risk among smokers, in particular former smokers. Although a residual confounding effect of smoking can not be completely ruled out, two reasons argue that our results are unlikely chance findings: (1) intake of individual cruciferous vegetables, especially consumed as raw, were significantly associated with reduction of lung cancer risk (results not shown); and (2) multiple lines of biological evidence provides plausible underlying mechanisms to support our observation. Considering the relatively larger sample size and better control of the confounding effect of smoking, our data supports that a diet high in cruciferous vegetables, particularly when consumed as raw, may reduce the risk of lung cancer among smokers. These findings, along with those of other investigators, indicate cruciferous vegetable may play a more important role in cancer prevention among people exposed to cigarette-smoking.

## Competing interests

The authors declare that they have no competing interests.

## Authors' contributions

LT and GRZ carried out the statistical analysis and drafted the manuscript. VJ, MER, SEM, CEN, YZ, CBA, and KBM participated in the design and coordination of the study. TL, SEM, CBA, and KBM conceived of the study. All authors read and approved the final manuscript.

## Pre-publication history

The pre-publication history for this paper can be accessed here:

http://www.biomedcentral.com/1471-2407/10/162/prepub

## Supplementary Material

Additional file 1 **Odds Ratios (OR) and 95% Confidence Intervals (CI) for the Association of Lung Cancer Risk with Fruit, Vegetable, and Cruciferous Vegetable Intake by Smoking Status**. Click here for file

Additional file 2 **Adjusted Odds Ratios (OR) and 95% Confidence Intervals (CI) for the Association of Lung Cancer Risk with Fruit, Vegetable, and Cruciferous Vegetable Intake by Number of Cigarettes per day and Years of Smoking**. Click here for file

Additional file 3 **Odds Ratios (OR) and 95% Confidence Intervals (CI) for the Association of Lung Cancer Risk with Fruit, Vegetable, and Cruciferous Vegetable Intake by Histological Subtypes**. Click here for file
