# Supplementary material for: Cruciferous vegetable intake is inversely associated with lung cancer risk among smokers: a case-control study
Source: BMC Cancer. 2010 Apr 27;10:162. doi: 10.1186/1471-2407-10-162 (PMC2874783; doi:10.1186/1471-2407-10-162)
Supplement: Additional file 1 — Odds Ratios (OR) and 95% Confidence Intervals (CI) for the Association of Lung Cancer Risk with Fruit, Vegetable, and Cruciferous Vegetable Intake by Smoking Status. [file 1471-2407-10-162-S1.DOC]

**Table 3. Odds Ratios (OR) and 95% Confidence Intervals (CI) for the Association of Lung Cancer Risk with Fruit, Vegetable, and Cruciferous Vegetable Intake by Smoking Status**

|  | Never Smoker | | | Former Smoker | | | Current Smoker | | |
| --- | --- | --- | --- | --- | --- | --- | --- | --- | --- |
|  | Cases | Controls | Adjusted OR^1^ (95% CI) | Cases | Controls | Adjusted OR^1^ (95% CI) | Cases | Controls | Adjusted OR^1^ (95% CI) |
| Vegetables, servings/mo | |  |  |  |  |  |  |  |  |
| <58 | 27 | 40 | 1.00 | 261 | 385 | 1.00 | 111 | 163 | 1.00 |
| 58-90 | 14 | 24 | 0.84 (0.36-1.98) | 233 | 446 | 0.83 (0.64-1.08) | 59 | 115 | 0.75 (0.49-1.15) |
| >90 | 10 | 36 | 0.36 (0.15-0.90) | 190 | 438 | 0.70 (0.53-0.92) | 43 | 96 | 0.66 (0.40-1.06) |
|  |  |  | *P = 0.0292* |  |  | *P = 0.0118* |  |  | *P = 0.0784* |
| Fruits, servings/mo |  |  |  |  |  |  |  |  |  |
| <29.5 | 18 | 21 | 1.00 | 271 | 377 | 1.00 | 118 | 192 | 1.00 |
| 29.5-58.5 | 18 | 35 | 0.55 (0.23-1.32) | 216 | 444 | 0.85 (0.66-1.10) | 62 | 109 | 1.13 (0.74-1.73) |
| >58.5 | 15 | 44 | 0.40 (0.16-1.00) | 197 | 448 | 0.80 (0.61-1.05) | 33 | 73 | 0.97 (0.57-1.65) |
|  |  |  | *P = 0.0585* |  |  | *P = 0.1145* |  |  | *P = 0.9753* |
| Cruciferous, servings/mo | |  |  |  |  |  |  |  |  |
| <7 | 19 | 34 | 1.00 | 278 | 405 | 1.00 | 112 | 167 | 1.00 |
| 7-16 | 17 | 36 | 0.88 (0.38-2.04) | 223 | 416 | 0.90 (0.67-1.16) | 54 | 115 | 0.72 (0.47-1.11) |
| >16 | 15 | 30 | 0.77 (0.32-1.84) | 182 | 448 | 0.71 (0.54-0.93) | 47 | 92 | 0.73 (0.46-1.17) |
|  |  |  | *P = 0.5637* |  |  | *P = 0.0105* |  |  | *P = 0.2329* |
| Raw cruciferous, servings/mo | |  |  |  |  |  |  |  |  |
| <2.5 | 26 | 38 | 1.00 | 341 | 479 | 1.00 | 133 | 191 | 1.00 |
| 2.5-4.5 | 12 | 31 | 0.63 (0.26-1.49) | 194 | 401 | 0.81 (0.63-1.05) | 38 | 101 | 0.50 (0.31-0.79) |
| >4.5 | 13 | 31 | 0.59 (0.25-1.37) | 149 | 389 | 0.69 (0.53-0.91) | 42 | 82 | 0.73 (0.45-1.18) |
|  |  |  | *P = 0.2905* |  |  | *P = 0.0136* |  |  | *P = 0.3074* |

^1^ Odds ratios and 95% confidence intervals were calculated with unconditional logistic regression adjusted for age (continuous), education level (<high school or >high school), gender (male or female), total meat intake (continuous), number of cigarettes per day (continuous), years of smoking (continuous), and year of admission (continuous).
