# Supplementary material for: Cruciferous vegetable intake is inversely associated with lung cancer risk among smokers: a case-control study
Source: BMC Cancer. 2010 Apr 27;10:162. doi: 10.1186/1471-2407-10-162 (PMC2874783; doi:10.1186/1471-2407-10-162)
Supplement: Additional file 2 — Adjusted Odds Ratios (OR) and 95% Confidence Intervals (CI) for the Association of Lung Cancer Risk with Fruit, Vegetable, and Cruciferous Vegetable Intake by Number of Cigarettes per day and Years of Smoking. [file 1471-2407-10-162-S2.DOC]

**Table 4. Adjusted Odds Ratios (OR) and 95% Confidence Intervals (CI) for the Association of Lung Cancer Risk with Fruit, Vegetable, and Cruciferous Vegetable Intake** by Number of Cigarettes per day and Years of Smoking

|  | ≤ 20 Cigarettes / day | | | > 20 Cigarettes / day | | | ≤ 30 years of Smoking | | | > 30 years of Smoking | | |
| --- | --- | --- | --- | --- | --- | --- | --- | --- | --- | --- | --- | --- |
|  | Case | Control | Adjusted OR^1^ (95% CI) | Case | Control | Adjusted OR^1^ (95% CI) | Case | Control | Adjusted OR^2^ (95% CI) | Case | Control | Adjusted OR^2^ (95% CI) |
| Vegetables, servings/mo | | |  |  |  |  |  |  |  |  |  |  |
| <58 | 143 | 338 | 1.00 | 229 | 209 | 1.00 | 91 | 272 | 1.00 | 281 | 276 | 1.00 |
| 58-90 | 114 | 371 | 0.80 (0.59-1.10) | 177 | 190 | 0.79 (0.58-1.08) | 70 | 323 | 0.68 (0.47-0.98) | 221 | 238 | 0.82 (0.63-1.07) |
| >90 | 121 | 368 | 0.86 (0.62-1.18) | 112 | 166 | 0.52 (0.36-0.73) | 48 | 326 | 0.47 (0.31-0.71) | 185 | 207 | 0.79 (0.60-1.06) |
|  |  |  | *P = 0.3990* |  |  | *P = 0.0002* |  |  | *P = 0.0004* |  |  | *P = 0.1181* |
| Fruits, servings/mo | | |  |  |  |  |  |  |  |  |  |  |
| <29.5 | 142 | 342 | 1.00 | 246 | 226 | 1.00 | 92 | 280 | 1.00 | 297 | 289 | 1.00 |
| 29.5-58.5 | 124 | 358 | 0.95 (0.70-1.30) | 154 | 195 | 0.86 (0.63-1.18) | 67 | 318 | 0.79 (0.55-1.15) | 211 | 234 | 0.87 (0.67-1.13) |
| >58.5 | 112 | 377 | 0.77 (0.56-1.07) | 118 | 211 | 0.91 (0.64-1.30) | 50 | 323 | 0.64 (0.43-0.96) | 179 | 198 | 0.80 (0.60-1.07) |
|  |  |  | *P = 0.1145* |  |  | *P =0.5623* |  |  | *P = 0.0324* |  |  | *P = 0.1262* |
| Cruciferous, servings/mo | | |  |  |  |  |  |  |  |  |  |  |
| <7 | 156 | 376 | 1.00 | 235 | 195 | 1.00 | 88 | 281 | 1.00 | 303 | 290 | 1.00 |
| 7-16 | 113 | 324 | 0.94 (0.69-1.28) | 163 | 207 | 0.70 (0.51-0.95) | 68 | 298 | 0.74 (0.51-1.06) | 209 | 232 | 0.83 (0.64-1.08) |
| >16 | 109 | 377 | 0.75 (0.55-1.03) | 120 | 163 | 0.62 (0.44-0.88) | 53 | 341 | 0.50 (0.34-0.75) | 175 | 199 | 0.80 (0.60-1.06) |
|  |  |  | *P = 0.0646* |  |  | *P = 0.0122* |  |  | *P = 0.0010* |  |  | *P = 0.1512* |
| Raw cruciferous, servings/mo | | |  |  |  |  |  |  |  |  |  |  |
| <2.5 | 194 | 413 | 1.00 | 280 | 256 | 1.00 | 104 | 328 | 1.00 | 370 | 342 | 1.00 |
| 2.5-4.5 | 96 | 339 | 0.66 (0.48-0.90) | 135 | 163 | 0.77 (0.56-1.06) | 64 | 293 | 0.65 (0.45-0.94) | 168 | 208 | 0.77 (0.59-1.01) |
| >4.5 | 88 | 325 | 0.69 (0.49-0.94) | 103 | 146 | 0.68 (0.48-0.95) | 41 | 300 | 0.45 (0.30-0.68) | 149 | 171 | 0.78 (0.59-1.04) |
|  |  |  | *P = 0.0575* |  |  | *P = 0.0381* |  |  | *P = 0.0004* |  |  | *P = 0.1480* |

^1^ Odds ratios and 95% confidence intervals were calculated with unconditional logistic regression adjusted for age (continuous), education level (<high school or >high school), gender (male or female), total meat intake (continuous), smoking status (never, quit, or current), years of smoking (continuous), and year of admission (continuous).

^2^ Odds ratios and 95% confidence intervals were calculated with unconditional logistic regression adjusted for age (continuous), education level (<high school or >high school), gender (male or female), total meat intake (continuous), smoking status (never, quit, or current), number of cigarettes per day (continuous), and year of admission (continuous).
