# Supplementary material for: Cruciferous vegetable intake is inversely associated with lung cancer risk among smokers: a case-control study
Source: BMC Cancer. 2010 Apr 27;10:162. doi: 10.1186/1471-2407-10-162 (PMC2874783; doi:10.1186/1471-2407-10-162)
Supplement: Additional file 3 — Odds Ratios (OR) and 95% Confidence Intervals (CI) for the Association of Lung Cancer Risk with Fruit, Vegetable, and Cruciferous Vegetable Intake by Histological Subtypes. [file 1471-2407-10-162-S3.DOC]

**Table 5. Odds Ratios (OR) and 95% Confidence Intervals (CI) for the Association of Lung Cancer Risk with Fruit, Vegetable, and Cruciferous Vegetable Intake by Histological Subtypes**

|  |  | Squamous  (N=347) | | Small Cell  (N=204) | | Large cell  (N=162) | | Adenocarcinoma  (N=120) | | Others  (N=115) | | Overall  (N=948) |
| --- | --- | --- | --- | --- | --- | --- | --- | --- | --- | --- | --- | --- |
|  | Control  (N=1743) | N | Adjusted OR*  (95% CI) | N | Adjusted OR*  (95% CI) | N | Adjusted OR*  (95% CI) | N | Adjusted OR*  (95% CI) | N | Adjusted OR*  (95% CI) | Adjusted OR*  (95% CI) |
| Vegetables, servings/mo | | |  |  |  |  |  |  |  |  |  |  |
| <58 | 588 | 153 | 1.00 | 99 | 1.00 | 71 | 1.00 | 37 | 1.00 | 39 | 1.00 | 1.00 |
| 58-90 | 585 | 103 | 0.66  (0.49-0.89) | 58 | 0.63  (0.44-0.90) | 47 | 0.66  (0.44-0.99) | 46 | 1.29  (0.81-2.04) | 52 | 1.37  (0.88-2.14) | 0.79  (0.64-0.98) |
| >90 | 570 | 91 | 0.58  (0.42-0.80) | 47 | 0.57  (0.39-0.86) | 44 | 0.66  (0.43-1.01) | 37 | 1.04  (0.63-1.71) | 24 | 0.68  (0.39-1.17) | 0.65  (0.52-0.82) |
|  |  |  | *P=0.0010* |  | *P=0.0056* |  | *P=0.0617* |  | *P=0.9830* |  | *P=0.1427* | *P=0.0003* |
| Fruits, servings/mo | | |  |  |  |  |  |  |  |  |  |  |
| <29.5 | 590 | 146 | 1.00 | 97 | 1.00 | 78 | 1.00 | 51 | 1.00 | 35 | 1.00 | 1.00 |
| 29.5-58.5 | 588 | 103 | 0.76  (0.56-1.03) | 61 | 0.86  (0.60-1.24) | 46 | 0.72  (0.48-1.07) | 40 | 0.86  (0.55-1.35) | 46 | 1.63  (1.02-2.62) | 0.87  (0.70-1.07) |
| >58.5 | 565 | 98 | 0.76  (0.55-1.04) | 46 | 0.76  (0.51-1.14) | 38 | 0.65  (0.42-1.01) | 29 | 0.63  (0.38-1.03) | 34 | 1.37  (0.82-2.30) | 0.77  (0.62-0.97) |
|  |  |  | *P=0.0862* |  | *P=0.1800* |  | *P=0.0485* |  | *P=0.0672* |  | *P=0.2572* | *P=0.0273* |
| Cruciferous, servings/mo | | | |  |  |  |  |  |  |  |  |  |
| <7 | 606 | 149 | 1.00 | 107 | 1.00 | 72 | 1.00 | 36 | 1.00 | 46 | 1.00 | 1.00 |
| 7-16 | 567 | 111 | 0.85  (0.63-1.14) | 57 | 0.66  (0.46-0.94) | 43 | 0.70  (0.47-1.05) | 44 | 1.31  (0.82-2.08) | 39 | 0.95  (0.61-1.50) | 0.83  (0.67-1.03) |
| >16 | 570 | 87 | 0.66  (0.48-0.91) | 40 | 0.48  (0.32-0.71) | 47 | 0.79  (0.52-1.18) | 40 | 1.15  (0.71-1.85) | 30 | 0.76  (0.46-1.24) | 0.70  (0.56-0.87) |
|  |  |  | *P=0.0137* |  | *P=0.0005* |  | *P=0.3726* |  | *P=0.7995* |  | *P=0.2499* | *P=0.0024* |
| Raw cruciferous, servings/mo | | |  |  |  |  |  |  |  |  |  |  |
| <2.5 | 708 | 199 | 1.00 | 122 | 1.00 | 77 | 1.00 | 51 | 1.00 | 51 | 1.00 | 1.00 |
| 2.5-4.5 | 533 | 77 | 0.58  (0.43-0.79) | 48 | 0.58  (0.40-0.84) | 44 | 0.86  (0.57-1.29) | 34 | 0.86  (0.54-1.36) | 41 | 1.10  (0.71-1.71) | 0.71  (0.58-0.88) |
| >4.5 | 502 | 71 | 0.58  (0.42-0.80) | 34 | 0.49  (0.32-0.74) | 41 | 0.90  (0.59-1.37) | 35 | 0.96  (0.61-1.53) | 23 | 0.72  (0.43-1.22) | 0.67  (0.54-0.84) |
|  |  |  | *P=0.0040* |  | *P=0.0023* |  | *P=0.7321* |  | *P=0.9982* |  | *P=0.1722* | *P=0.0023* |

^*^ Odds ratios and 95% confidence intervals were calculated with unconditional logistic regression adjusted for age (continuous), education level (<high school or >high school), gender (male or female), total meat intake (continuous), smoking status (never, quit, or current), number of cigarettes per day (continuous), years of smoking (continuous), and year of admission (continuous).
